# Heat stress resistance in *Camelina sativa*: effects on morphology, physiology, yield, and seed quality

**DOI:** 10.3389/fpls.2025.1719558

**Published:** 2025-12-04

**Authors:** Barbara Alberghini, Federica Zanetti, Federico Ferioli, Richard P. Haslam, Susana Silvestre, Andrea Monti

**Affiliations:** 1 Department of Agricultural and Food Sciences (DISTAL), Alma Mater Studiorum – Università di Bologna, Bologna, Italy; 2 Rothamsted Research, Harpenden, United Kingdom

**Keywords:** fatty acids, heat, oil content, oilseed crops, seed yield, tocopherols

## Abstract

Cultivated crops are increasingly exposed to episodes of extreme heat. In the Mediterranean basin, crops often experience heat stress during spring or summer, coinciding with flowering and seed ripening. Recently, *Camelina sativa* has emerged as an alternative oilseed crop of interest due to its resistance to abiotic stresses. To investigate possible mechanisms underlying camelina’s ability to cope with heat stress and to evaluate the role of tocopherols, two spring varieties (Cypress and Omega) were tested in two controlled-environment experiments. Heat was imposed for five consecutive days either from the end of flowering (EXP1) or from the stage when siliques reached their final size (EXP2). Early imposition of heat stress (EXP1) had the greatest impact on camelina morphological parameters during the growth cycle. At harvest in EXP1, only the genotype significantly affected plant height and seed yield, with Omega producing taller plants and higher seed yield (0.83 g per plant) compared with Cypress (0.70 g per plant). In EXP2, cultivar significantly affected only straw weight, which was higher in Omega. Nonetheless, Cypress exhibited the highest 1,000-seed weight in both experiments (1.36 g in EXP1 and 1.34 g in EXP2). Seed oil content was reduced by heat stress (− 9.89% in EXP1 and − 11.6% in EXP2, respectively). Fatty acid composition in EXP1 was mainly influenced by the cultivar, except for C18:1, whereas in EXP2, heat stress predominantly affected 18-carbon fatty acids. Total tocopherol content was largely under genetic control, and although α-tocopherol is associated with responses to abiotic stress, it increased only when stress was imposed at a later stage (+ 75.8% in the stressed plants). Despite the high tocopherol content of camelina, it appeared to contribute to plant stress resistance only under late-stage heat stress dueing seed maturation.

## 1 Introduction

In recent years, global climate change has become a major concern for agricultural researchers and producers alike. The Mediterranean basin, in particular, has been highlighted as a hotspot (Lazoglou et al., 2024) due to its increased susceptibility to climate change. In particular, extreme temperature events, such as heat waves and days with maximum temperatures above 37°C, have increased in recent decades and are expected to become more frequent in the coming years (Bilalis et al., 2017; Intergovernmental Panel On Climate Change (Ipcc), 2023). Elevated temperatures affect crops by impacting their phenology and shortening the growing season, collectively reducing yields on farms (Hasanuzzaman et al., 2013; Intergovernmental Panel On Climate Change (Ipcc), 2023; Kaushal et al., 2016). Consequently, climate change is likely to continue and exert an even greater impact on agricultural production in the Mediterranean region.

High-temperature stress, or heat stress, occurs when a rise in temperature exceeds a critical threshold and lasts long enough to impair plant development (Hasanuzzaman et al., 2013; Wahid et al., 2007). The agronomic impact of heat stress depends on the developmental stage at which it occurs, with the reproductive phase often being highly susceptible. This can have detrimental effects on yield, as the optimal temperature for this phenological stage is lower than that for vegetative development (Hatfield et al., 2011; Prasad et al., 2017). In this context, a plant that can maintain seed production despite heat stress may be considered heat-tolerant (Źróbek-Sokolnik, 2012). Within this framework, alternative crops that can tolerate heat stress and adapt to warming European and Mediterranean environments are of high value, making it essential to investigate how this species can mitigate the effects of heat stress (Wang et al., 2025). Camelina (*Camelina sativa* L. Crantz) has recently been rediscovered and is now proposed as a viable option (Berzuini et al., 2024; Kakabouki et al., 2021; Zanetti et al., 2021) due to its agronomic and seed oil characteristics, as well as the availability of diverse spring varieties adaptable to a summer harvest. Moreover, camelina has been reported to exhibit resistance to diseases and pests typical of the Brassicaceae family (Séguin-Swartz et al., 2009) and to various abiotic stresses (Alberghini et al., 2025). Camelina seed contains 30% to 40% oil by dry weight and is rich in polyunsaturated fatty acids, particularly linoleic (C18:2) and linolenic acids (C18:3), which together account for more than 50% of the seed oil’s fatty acids. Furthermore, camelina seed oil is characterized by a noticeable tocopherol content, ranging from 675 to 1,565 mg kg^−1^ of oil (Abramovič et al., 2007; Günç Ergönül and Aksoylu Özbek, 2018; Hrastar et al., 2012).

Accumulation of tocopherols in seed oils is important because they belong to the vitamin E family of antioxidants. They occur in natural tissues in four different isoforms—α-, β-, δ-, and γ-tocopherol—depending on the number and positions of methyl groups in the chromanol head group (Hunter and Cahoon, 2007), and act as stabilizing agents for cell membranes (Falk and Munné-Bosch, 2010). Under oxidative stress, tocopherols act as antioxidants by both controlling the propagation of lipid peroxidation and scavenging singlet oxygen (^1^O_2_). In the first case, tocopherols protect polyunsaturated fatty acids (PUFAs, e.g., C18:3) from oxidation by donating a hydrogen atom to peroxy radicals before the radicals can extract it from PUFAs, thereby interrupting lipid peroxidation. The second type of antioxidant activity is necessary when excessive amounts of ^1^O_2_ are produced during stress, as its accumulation must be reduced to prevent cellular damage. This occurs through α-tocopherol, which can either physically quench ^1^O_2_ in chloroplasts or chemically react with it (Mène-Saffrané and DellaPenna, 2010; Munné-Bosch, 2005; Sattler et al., 2004). Tocopherol isoforms differ in their antioxidant activity in plants, with α-tocopherol being the most active (Munné-Bosch and Alegre, 2002). Beyond their role in plant tissues, tocopherols are also important components of vegetable oils obtained from seed pressing. They remain active as antioxidants, protecting the oil from oxidation (Choe and Min, 2006; Zaunschirm et al., 2018), and are essential for human health (Ju et al., 2010; Niu et al., 2022).

The aim of this work was to investigate the impact of heat stress in the reproductive stages of camelina and its consequences on yield, seed oil quality, and tocopherol content. To achieve this, two spring camelina varieties were subjected to heat stress imposed at two distinct phases of the plant reproductive period. Morphological and physiological parameters were monitored throughout the growing cycle, and at harvest, both quantitative and compositional properties of the seeds were determined. Particular attention was given to the fatty acid and tocopherol composition in the oil.

## 2 Materials and methods

### 2.1 Trial setup and management

Two growth chamber experiments were conducted at the Department of Agricultural and Food Sciences, University of Bologna, Italy. The two spring camelina cultivars, Cypress (Smart Earth, Saskatoon, Canada) and Omega (Poznan University, Poznan, Poland), originated from different breeding programs and were selected for distinctive traits (i.e., seed yield potential, seed size, tocopherol content). In each experiment, forty 100 mm × 100 mm × 120 mm pots were filled with turf-based potting soil (Vigor Plant Italia Srl, Fombio (LO), Italy). Twenty pots per cultivar were sown with five seeds per pot, arranged in a quincunx pattern at a depth of 5 mm. Germination and growth were performed under control conditions in a growth chamber at 25°C/18°C day/night (light intensity and photoperiod length: 400 μmol m^−2^ s^−1^ of PAR; 12 h; relative humidity: 60%). From the fifth fully expanded leaf stage (BBCH 105, according to Martinelli and Galasso, 2011), only one plant per pot was maintained. Water was applied once a week until the beginning of stem elongation and then twice a week at a volume equivalent to 30% of soil weight (g). In the first experiment (EXP1), the day temperature was increased to 35°C (18°C night) for five consecutive days, starting from the end of flowering (BBCH 607) on 10 randomly chosen pots per cultivar. The remaining 10 pots were maintained under control conditions (25/18°C day/night). In the second experiment (EXP2), day temperature was increased to 40°C (18°C night) for five consecutive days, starting from BBCH 709 (when almost all siliques had reached their final size) on 10 randomly chosen pots per cultivar. The remaining 10 pots were kept under control conditions (25°C/18°C day/night). These high-temperature settings were used to simulate field conditions that can occur in spring in Northern Italy, as previously reported by Righini et al. (2019). During heat stress treatment, plants were watered daily to prevent water stress. In both experiments, plants were returned to control temperature (25°C/18°C) after 5 days of high temperature and allowed to mature and set seed.

### 2.2 Measurements during the growth cycle

Morphological parameters (plant height in cm, number of lateral branches, and number of leaves) and physiological parameters (chlorophyll content and gas exchange) were used to assess plant performance. These measurements were taken the day before the temperature increase and after 24 h of recovery. The difference between values measured before and after high temperature imposition was calculated to obtain the net variation (Δ) of each measured parameter, allowing a more precise evaluation of the intensity of the response to high temperature.

Chlorophyll content was measured on the seventh fully expanded leaf using a SPAD-502 (Minolta Co. Ltd., Japan). Gas exchange parameters were assessed on siliques of the main raceme using the portable photosynthesis system CIRAS-3 (PP Systems, Amesbury, MA, USA) with a PLC3 conifer and narrow cuvette equipped with an LED light unit. The measured parameters were net photosynthesis (*A*, µmol m^−2^ s^−1^) and stomatal conductance (*g_s_*, mmol m^−2^ s^−1^).

### 2.3 Measurements at harvest

When plants reached physiological maturity, their height, number of siliques, and number of lateral branches were measured. Plants were then cut at soil level, seeds were manually separated by straw, and each part was weighed to determine net fresh weight (g). Representative subsamples of straw were dried at 60 °C until constant weight and then re-weighed. A sample of seeds from each treatment was dried at 105 °C until constant weight and then weighed again to determine seed dry matter. All straw and seed data were reported on a dry matter (DM) basis. To determine seed weight, 50 seeds from five plants per treatment were weighed, and the 1,000-seed weight (g) was then calculated.

### 2.4 Seed quality analysis

#### 2.4.1 Reagents and chemicals

Chemicals and solvents were of analytical grade (Merck, Darmstadt, Germany). Solvents used for high-performance liquid chromatography (HPLC) analysis, both for mobile phase and cleaning solution preparation, were of chromatographic grade. Deionized water was obtained using an Elix^®^ Essential 5 water purification system (Merck Millipore, Molsheim, France).

#### 2.4.2 Oil extraction from camelina seeds

About 0.5–1 g of camelina seeds was finely ground using a mortar. An aliquot of 0.2 g of ground material was precisely weighed into a 15-mL polypropylene (PP) centrifuge tube and subjected to the extraction procedure reported by Hara and Radin (1978), as described below. Ground seeds were mixed with 4 mL of *n*-hexane/*i*-propanol 3/2 (v/v), shaken on a vortex mixer for 10 s, incubated on a horizontal shaker for 5 min (motion speed: 640 m min^−1^), and then centrifuged at 2,647*×g* for 5 min at room temperature. The supernatant fraction was transferred to a second 15-mL PP centrifuge tube, while the residue was extracted and centrifuged twice more with 2 mL *n*-hexane/*i*-propanol 3/2 (v/v) each time. To remove nonlipidic material, the collected extracts were mixed with 4 mL of a 6.67% (w/w) aqueous solution of anhydrous Na_2_SO_4_, shaken on a vortex mixer for 10 s, and centrifuged at 2,647*×g* for 3 min. The supernatant fraction was transferred to and stored in a third 15-mL PP centrifuge tube at − 18°C until further analyses.

#### 2.4.3 Determination of oil content

Organic extracts were thawed at room temperature for 10–15 min and then transferred to a 100-mL, previously weighed flat-bottom flask. Solvent removal was carried out using a rotary evaporator under reduced pressure (bath temperature: 30°C). The recovered oil was dried under a nitrogen gas flow for 2 min in a water bath maintained at about 50°C–55°C, weighed, transferred to a glass conical tube with two aliquots (1 mL each) of *n*-hexane/*i*-propanol 4/1 (v/v), and stored at − 18°C until further analyses.

#### 2.4.4 Determination of tocopherols by high-performance liquid chromatography coupled with fluorometric detection

Tocopherol determination was performed in isocratic mode on an HPLC system from Jasco (Tokyo, Japan) equipped with two binary pumps (mod. PU-1580), a fluorescence detector (mod. FP-1520), and an autosampler (mod. AS-2055 Plus). The mobile phase was the same as that used by Panfili et al. (2003): *n*-hexane/ethyl acetate/acetic acid (97.3:1.8:0.9, v/v/v), whereas *n*-hexane/*i*-propanol (99/1 v/v) was used as a cleaning solution for the autosampler syringe before and after sample injection. Both the mobile phase and cleaning solution were preliminary degassed in an ultrasonic bath for 15 min at room temperature.

Flow rate and injection volume were set at 1.5 mL min^−1^ and 20 μL, respectively. HPLC traces were acquired in 20 min. Fluorometric detection was performed at an excitation wavelength of 290 nm and an emission wavelength of 330 nm. A Kromasil 100-5SIL column (250 mm × 4.6 mm i.d., 5.0 μm particle size) from Eka Chemicals AB (Bohus, Sweden) was used for compound separation and equipped with a guard cartridge HILIC (4 mm × 2.0 mm i.d.) from Phenomenex (Torrance, CA, USA). Column temperature was maintained at 30°C during analyses. Tocopherols were identified and quantified using an external standard method with mixtures of α-, γ-, and δ-tocopherol standards. Stock solutions of each compound were prepared in *n*-hexane/*i*-propanol (99/1 v/v) at the following concentrations: 2.0700, 2.4185, and 2.0864 mg mL^−1^ for α-, γ-, and δ-tocopherol, respectively. Diluted mixtures for calibration curves covered the following concentration ranges: 0.0010–0.0250 (five calibration points, *r*^2^ > 0.99), 0.0005–0.0484 (seven calibration points, *r*^2^ > 0.99), and 0.0005–0.0098 (five calibration points, *r*^2^ > 0.99), for α-, γ-, and δ-tocopherol, respectively. Before HPLC injection, about 20 mg of oil, dissolved in *n*-hexane/*i*-propanol, was dried under a nitrogen flow in a previously weighed glass conical tube placed in a thermal heater set at 40°C until constant weight, and redissolved in 2 mL of *n*-hexane/*i*-propanol (99/1 v/v). Oil samples and standard compound solutions were filtered into HPLC amber glass vials through regenerated cellulose (RC) syringe filters (diameter: 13 mm, pore dimension: 0.45 μm) from GVS Filter Technology (Indianapolis, IN, USA) and analyzed in duplicate on an HPLC system. A blank was performed by injecting *n*-hexane/*i*-propanol (99/1 v/v) every 10 injections. Data were processed using ChromNAV (ver.1.16.02) from Jasco.

#### 2.4.5 Determination of fatty acids

Fatty acids were converted to methyl esters (FAME) and successively analyzed by gas chromatography with flame ionization detection (GC-FID) after a cold transmethylation procedure performed on camelina oil, according to Christopherson and Glass (1969). Transmethylation and GC operating conditions were carried out as previously described (Zanetti et al., 2022).

### 2.5 Statistical analysis

Prior to analysis of variance (ANOVA), which was performed separately for each experiment, the homoscedasticity of variance was verified using Bartlett’s test for *p* ≤ 0.05. A one-way ANOVA, considering cultivar as the main factor, was conducted for measurements taken before heat stress. For all the other data, a two-way ANOVA was performed, with cultivar and treatment as main factors. When ANOVA results were significant, the LSD test was applied to separate means (*p* ≤ 0.05). Statistical analyses were carried our using COSTAT software, version 6.204 (CoHortSoftware, USA).

## 3 Results

### 3.1 Morphological and physiological parameters during the growth cycle

**Figure 1:**
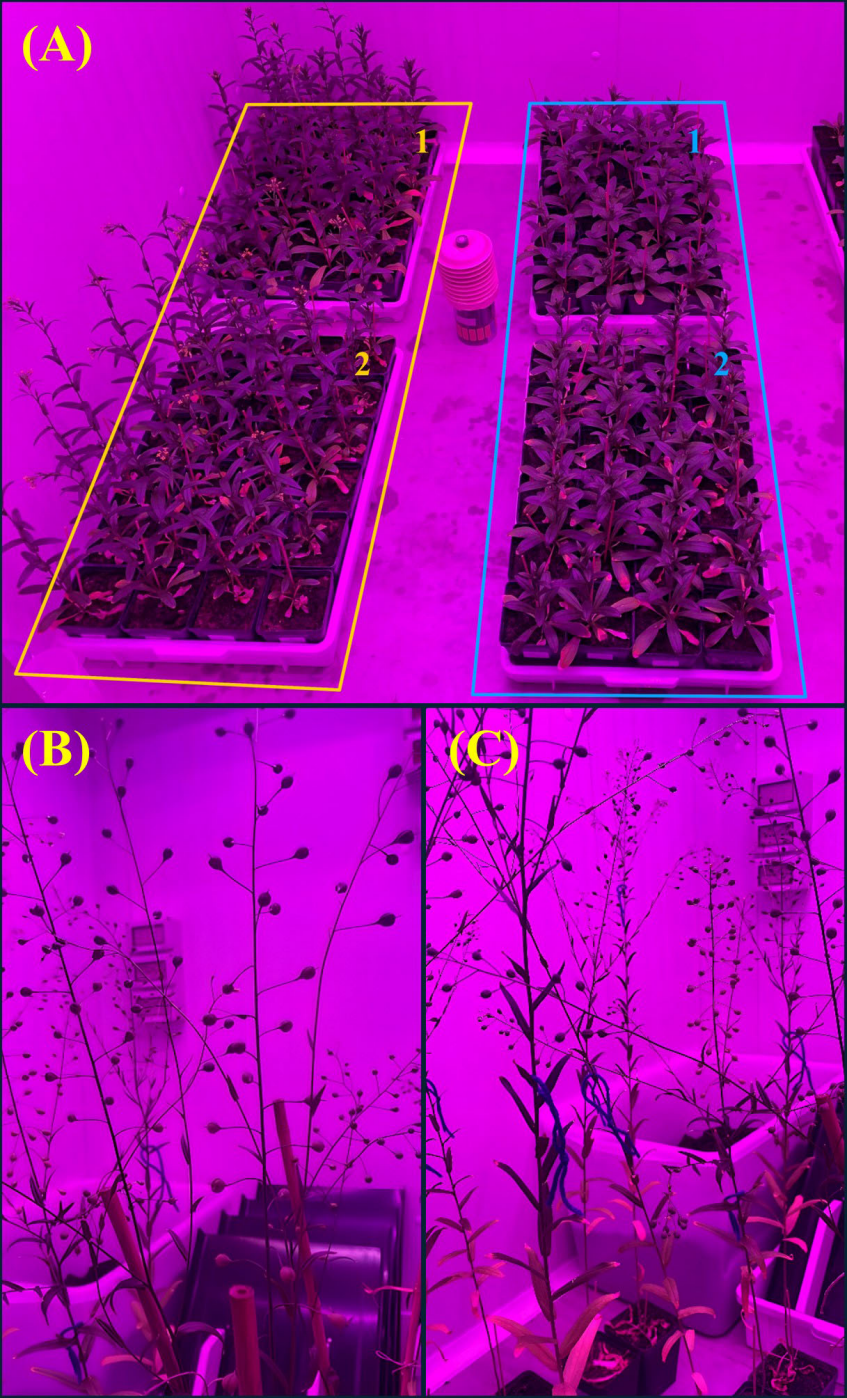
**(A)** Plants of EXP1 (yellow border) and EXP2 (blue border), belonging to cultivar Cypress (1) and Omega (2). **(B)** Siliques of EXP1 control plants. **(C)** Siliques of EXP1 stressed plants after heat treatment.

To assess the impact of heat stress on plant performance and yield, two spring camelina cultivars (Cypress and Omega) were subjected to two regimes in which stress was applied either at the end of flowering (EXP1) or the end of fruit development (EXP2), mimicking potential field conditions (**Figure 1A**). In EXP1, prior to stress imposition, Omega had significantly (*p* ≤ 0.05) taller plants with a higher number of leaves, while Cypress exhibited higher leaf chlorophyll content (**Table 1**). After heat stress, the main factor “cultivar” significantly (*p* ≤ 0.05) influenced silique stomatal conductance, which was higher in Cypress (410.6 mmol m^−2^ s^−1^) than in Omega (252.1 mmol m^−2^ s^−1^). Regarding the specific effect of heat treatment, significance (*p* ≤ 0.05) was observed only for net photosynthesis (**Table 2**). Stressed plants had lower net photosynthesis (19.7 µmol m^−2^ s^−1^) compared with the control (31.9 µmol m^−2^ s^−1^). The interaction “cultivar × treatment” was significant (*p* ≤ 0.05) for plant height and leaf chlorophyll content. Despite generally smaller size, Cypress was less affected by high temperature than Omega; indeed, Cypress showed no significant difference in plant height between in treated (72.8 cm) and control plants (72.9 cm). Regarding chlorophyll content, Cypress had a higher SPAD value (51.7) than Omega (46.0). Heat reduced chlorophyll content in both cultivars; however, the decrease was less pronounced in Cypress (from 52.6 in control to 50.7 in stressed plants) than in Omega (from 48.3 in control to 43.7 in stressed plants). Regarding net variation, the main factor cultivar was significant (*p* ≤ 0.05) for the number of lateral branches, number of leaves, chlorophyll content, and stomatal conductance (**Table 3**). For all these parameters, net variation was higher in Cypress than in Omega. Indeed, net variation values were as follows: number of lateral branches Δ = 2.29, number of leaves Δ = − 3.64, chlorophyll content Δ = − 3.14, and stomatal conductance Δ = 109.3. Although lower, net variation in Omega showed the same trends as in Cypress: the number of lateral branches increased (Δ = 0.10), the number of leaves decreased (Δ = − 6.40), and chlorophyll content decreased (Δ = − 6.42). Conversely, in Cypress, stomatal conductance increased, whereas in Omega it decreased, showing a net variation of − 72.8. The main factor, “treatment”, significantly (*p* ≤ 0.05) influenced the net variation for the number of leaves, stomatal conductance, and net photosynthesis. All plants lost leaves during heat stress; however, control plants exhibited a less pronounced reduction (Δ = − 3.59) compared with stressed plants (Δ = − 6.94). Heat increased stomatal conductance (Δ = 77.1), whereas it decreased in the control (Δ = − 72.8). The opposite trend was observed for net photosynthesis, which decreased under high temperature (Δ = − 5.28) and increased in the control (Δ = 6.44).

**Table 1:** Parameters measured before heat stress imposition in EXP1 and in EXP2.

Main factor	Plant height (cm)	No. of lateral branches	No. of leaves	Chlorophyll content (SPAD index)	*g_s_* (mmol m^−2^ s^−1^)	*A* (µmol m^−2^ s^−1^)
EXP1						
Cultivar						
Cypress	66.1 ± 0.54 b	5.07 ± 0.44 a	20.1 ± 0.73 b	54.8 ± 0.87 a	301.3 ± 25.2 a	25.6 ± 1.19 a
Omega	71.4 ± 1.64 a	6.55 ± 0.59 a	31.7 ± 0.94 a	52.4 ± 0.58 b	324.9 ± 40.7 a	24.9 ± 1.59 a
EXP2						
Cultivar						
Cypress	83.7 ± 1.48 b	4.10 ± 0.60 a	10.7 ± 0.44 b	19.9 ± 2.06 b	404.6 ± 20.3 a	15.5 ± 0.60 a
Omega	89.9 ± 2.39 a	3.55 ± 0.46 a	24.0 ± 1.27 a	33.1 ± 1.44 a	175.0 ± 7.47	15.2 ± 1.16 a

Mean value ± standard error. Different letters: statistically different means for *p* ≤ 0.05 (LSD Fisher’s test) within the same parameter, experiment, and main experimental factor.

**Table 2:** Parameters measured after heat stress imposition in EXP1 and in EXP2.

Main factor	Plant height (cm)	No. of lateral branches	No. of leaves	Chlorophyll content (SPAD index)	*g_s_* (mmol m^−2^ s^−1^)	*A* (µmol m^−2^ s^−1^)
EXP1						
Cultivar						
Cypress	72.3 ± 0.76 b	7.36 ± 0.80 a	16.5 ± 0.56 b	51.7 ± 0.76 a	410.6 ± 58.5 a	25.2 ± 2.64 a
Omega	78.5 ± 1.87 a	6.65 ± 0.63 a	25.3 ± 1.12 a	46.0 ± 0.83 b	252.1 ± 18.2 b	26.2 ± 1.88 a
Stress						
Control	78.3 ± 1.72 a	6.41 ± 0.63 a	22.9 ± 1.69 a	49.3 ± 0.67 a	327.8 ± 38.3 a	31.9 ± 1.39 a
Heat	73.6 ± 1.68 b	7.47 ± 0.76 a	20.4 ± 1.11 a	47.4 ± 1.32 a	307.0 ± 45.1 a	19.7 ± 1.73 b
EXP2						
Cultivar						
Cypress	83.8 ± 1.46 b	3.50 ± 0.37 a	2.62 ± 0.58 b	5.06 ± 1.64 b	246.4 ± 39.2 a	3.48 ± 0.53 b
Omega	90.2 ± 2.41 a	3.40 ± 0.29 a	5.31 ± 1.19 a	18.2 ± 2.22 a	219.0 ± 24.4 a	5.58 ± 0.81 a
Stress						
Control	86.8 ± 1.90 a	3.00 ± 0.37 a	12.4 ± 1.57 a	11.6 ± 2.31 a	259.9 ± 46.5 a	5.24 ± 0.90 a
Heat	87.2 ± 2.32 a	3.90 ± 0.30 a	8.60 ± 1.44 b	11.7 ± 2.60 a	209.9 ± 15.6 a	3.78 ± 0.45 a

Mean value ± standard error. Different letters: statistically different means for *p* ≤ 0.05 (LSD Fisher’s test) within the same parameter, experiment, and main experimental factor.

**Table 3:** Delta values calculated for the measured parameters in EXP1 and in EXP2.

Main factor	Plant height (Δ)	No. of lateral branches (Δ)	No. of leaves (Δ)	Chlorophyll content (Δ)	*g_s_* (Δ)	*A* (Δ)
EXP1						
Cultivar						
Cypress	6.18 ± 0.64 a	2.29 ± 0.60 a	− 3.64 ± 0.64 a	− 3.14 ± 0.58 a	109.3 ± 65.9 a	− 0.42 ± 2.56 a
Omega	7.13 ± 1.33 a	0.10 ± 0.07 b	− 6.40 ± 0.74 b	− 6.42 ± 0.59 b	− 72.8 ± 38.5 b	1.28 ± 1.92 a
Stress						
Control	7.35 ± 1.11 a	0.94 ± 0.37 a	− 3.59 ± 0.64 a	− 4.29 ± 0.61 a	− 72.8 ± 53.7 b	6.44 ± 1.38 a
Heat	6.12 ± 1.22 a	1.06 ± 0.50 a	− 6.94 ± 0.71 b	− 5.84 ± 0.77 a	77.1 ± 49.0 a	− 5.28 ± 1.86 b
EXP2						
Cultivar						
Cypress	0.15 ± 0.15 a	− 0.60 ± 0.44 a	− 5.7 ± 0.50 a	− 14.8 ± 2.09 a	− 154.9 ± 42.4 b	− 11.8 ± 0.72 a
Omega	0.30 ± 0.30 a	− 0.15 ± 0.11 a	− 7.95 ± 1.06 b	− 14.9 ± 1.99 a	52.5 ± 23.4 a	− 11.1 ± 0.99 a
Stress						
Control	0.45 ± 0.33 a	− 0.20 ± 0.17 a	− 4.55 ± 0.34 a	− 15.4 ± 1.79 a	− 32.8 ± 48.2 a	− 9.84 ± 0.84 a
Heat	0.00 ± 0.00 a	− 0.55 ± 0.41 a	− 9.10 ± 0.91 b	− 14.3 ± 2.26 a	− 87.5 ± 36.5 a	− 13.0 ± 0.70 b

Mean value ± standard error. Different letters: statistically different means for *p* ≤ 0.05 (LSD Fisher’s test) within the same parameter, experiment, and main experimental factor.

In EXP2, prior to heat stress, Omega again showed significantly (*p* ≤ 0.05) taller plants, with more leaves and higher chlorophyll content compared with Cypress, which, in contrast, exhibited higher silique stomatal conductance (**Table 1**). For plant height, number of leaves, and chlorophyll content, the same behavior was observed even after heat stress, with Omega maintaining higher values than Cypress (**Table 2**). Moreover, the main factor “treatment” significantly (*p* ≤ 0.05) influenced the number of leaves, which was higher in the control (12.4) than in the stressed plants (8.60). Net variation of the number of leaves was significantly (*p* ≤ 0.05) affected by factor cultivar, “treatment”, and their interaction (cultivar × treatment) (**Table 3**). Both cultivars lost leaves under stress; however, in Cypress, this change (Δ = − 4.5 in control, Δ = − 6.9 in stressed plants) was less pronounced than in Omega (Δ = − 4.6 in control, Δ = − 11.3 in stressed plants). The main factor cultivar significantly (*p* ≤ 0.05) influenced net variation in silique stomatal conductance, which increased in Omega (Δ = 42.5) but decreased in Cypress (Δ = − 187.7). Finally, the main factor “treatment” was significant (*p* ≤ 0.05) for net variation in net photosynthesis of siliques, which decreased in both treatments, with a sharper decline in stressed plants (Δ = − 9.84 in control, Δ = − 13.0 in stressed plants) (**Table 3**).

### 3.2 Performance at harvest

At harvest in EXP1, only the number of siliques was significantly (*p* ≤ 0.05) affected by treatment, with stressed plants producing more siliques (86) than controls (75.5) (**Figures 1B, C**; **Table 4**). The number of siliques was also influenced by cultivar, along with straw weight, seed yield, and 1,000-seed weight. Omega had more siliques (89.0), higher straw weight (1.27 g), and greater seed yield (0.83 g) than Cypress (72.6, 1.02, and 0.70 g, respectively). However, Cypress had a higher 1,000-seed weight (1.36 g) compared with Omega (0.92 g). Finally, plant height was affected (*p* ≤ 0.05) by the interaction cultivar × treatment, with control plants of Omega being significantly taller (78.3 cm) than all others.

**Table 4:** ANOVA table with *F*-values and statistical significance for parameters measured at harvest.

Source of variation	Plant height	No. of lateral branches	No. of siliques	Straw weight	Seed weight	1,000-seed weight
EXP1						
Cultivar	12.6^**^	0.01 ns	10.9^**^	12.1^**^	7.33^*^	46.4^**^
Treatment	3.46 ns	0.41 ns	4.47 *	0.11 ns	0.01 ns	0.16 ns
Cultivar × treatment	5.07^*^	2.22 ns	0.53 ns	0.40 ns	0.20 ns	0.78 ns
EXP2						
Cultivar	3.50 ns	1.49 ns	0.30 ns	18.0^**^	2.69 ns	44.6^**^
Treatment	0.00 ns	5.97 *	2.82 ns	0.29 ns	0.09 ns	0.05 ns
Cultivar × treatment	1.06 ns	0.66 ns	0.12 ns	1.50 ns	0.09 ns	1.28 ns

^*^0.05 and ^**^0.01 indicate probability levels of significance (LSD Fisher’s test). *ns*, not significant.

In EXP2, only the main factor cultivar was significant (**Table 4**) for straw weight and 1,000-seed weight. Omega had higher straw weight per plant (1.02 g) compared with Cypress (0.72 g), while Cypress exhibited the highest 1,000-seed weight (1.34 g) compared with Omega (0.84 g).

### 3.3 Seed oil content and composition

**Figure 2:**
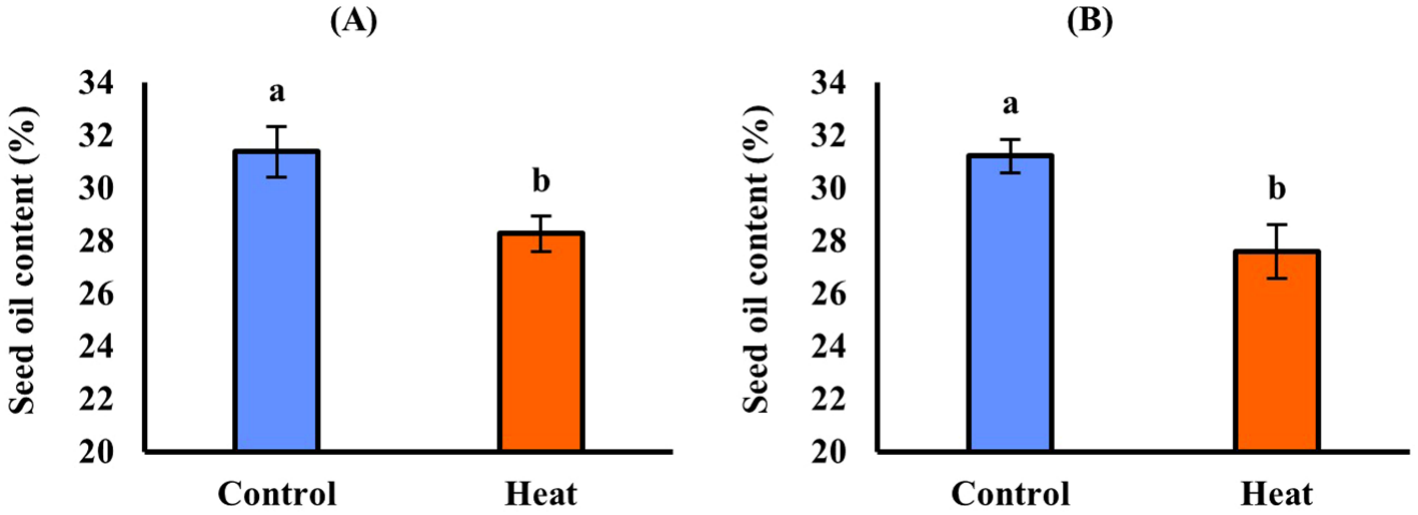
Seed oil content in response to treatment in EXP1 **(A)** and EXP2 **(B)**. Vertical bars: standard error. Different letters: significantly different means (*p* ≤ 0.05, LSD Fisher’s test).

**Figure 3:**
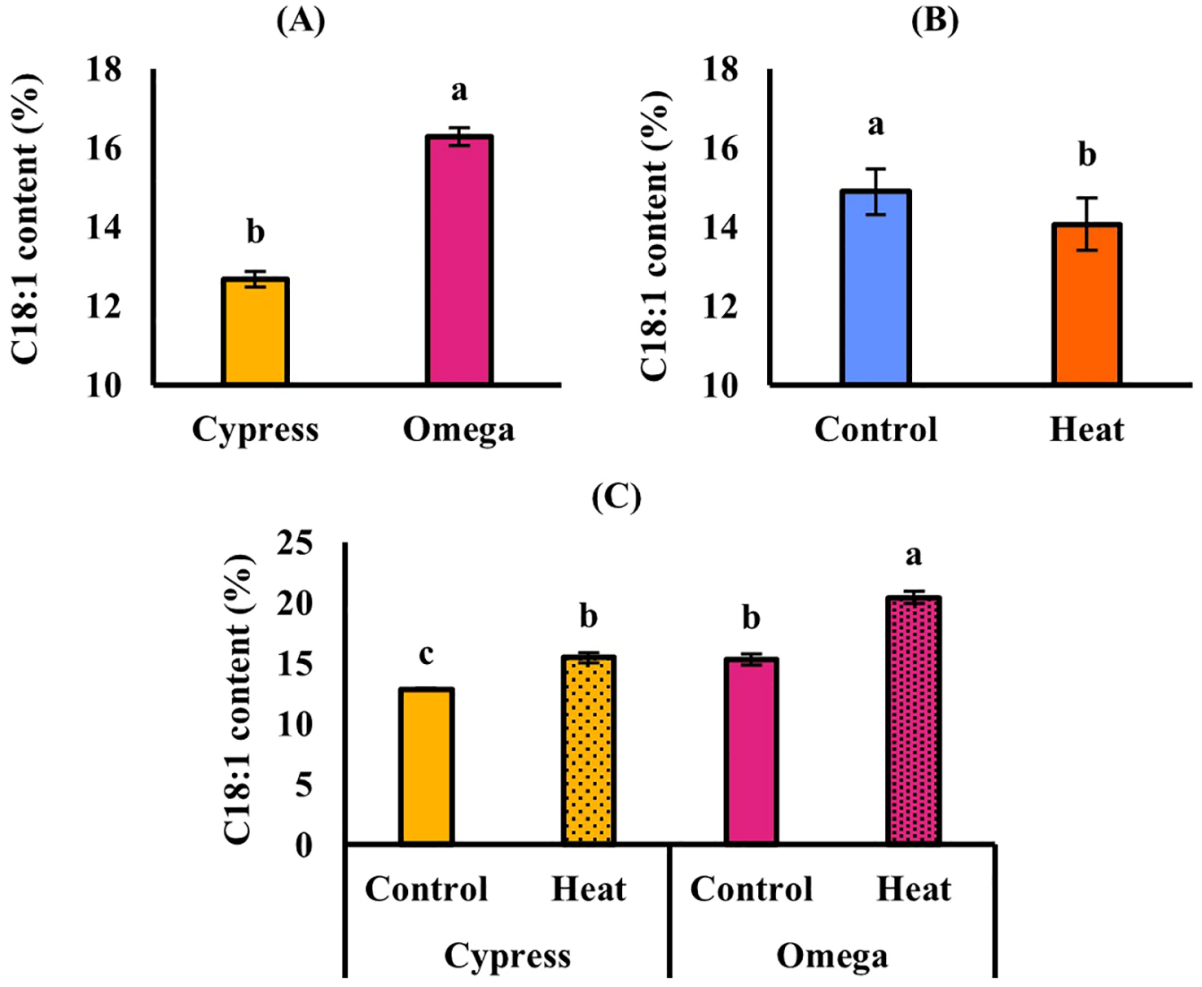
C18:1 content in response to cultivar in EXP1 **(A)**; to treatment in EXP1 **(B)**; and in response to “cultivar × treatment” interaction in EXP2. Vertical bars: standard error. Different letters: significantly different means (*p* ≤ 0.05, LSD Fisher’s test).

**Figure 4:**
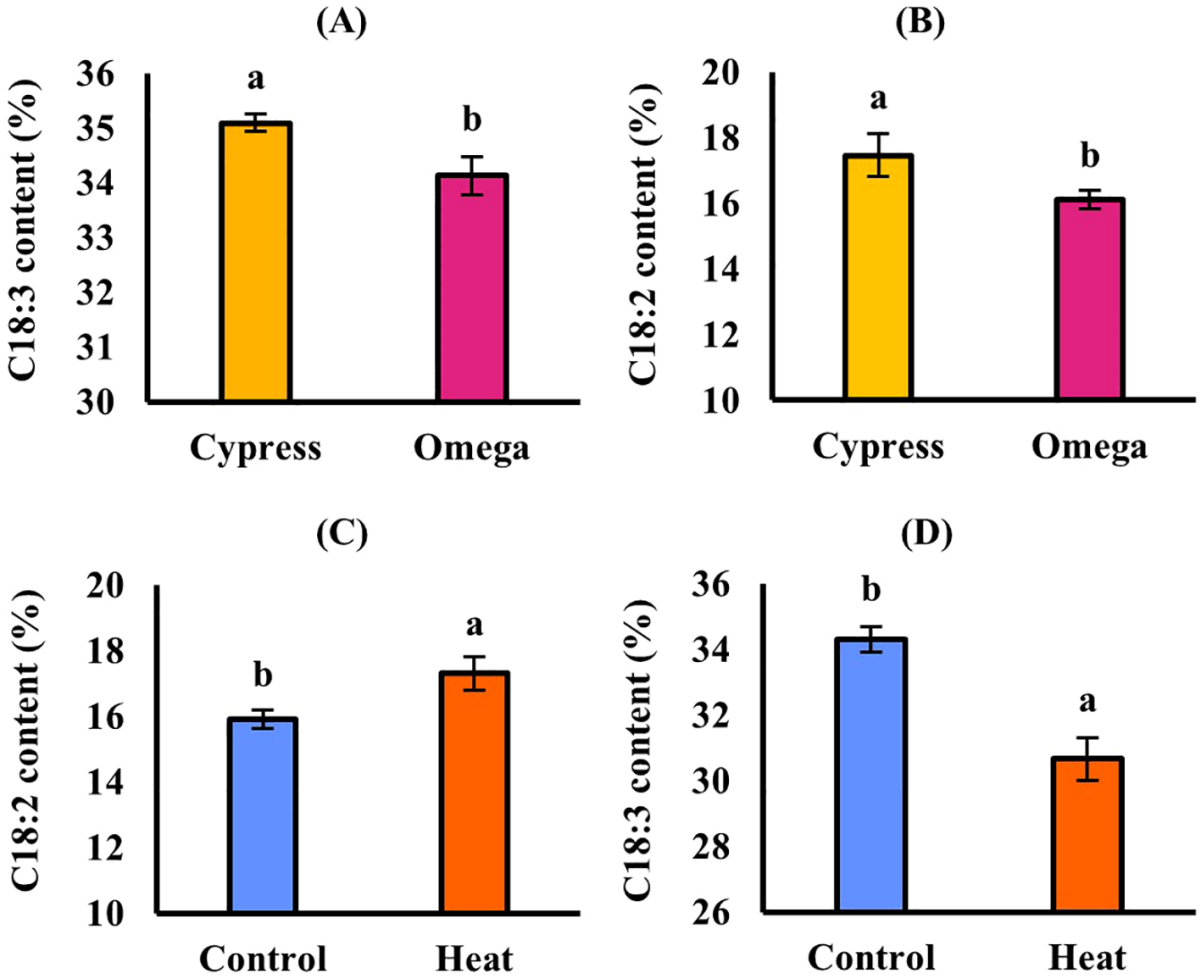
C18:3 content in response to cultivar in EXP1 **(A)**; C18:2 in response to cultivar in EXP2 **(B)**; C18:2 **(C)** and C18:3 **(D)** in response to treatment in EXP2. Vertical bars: standard error. Different letters: significantly different means (*p* ≤ 0.05, LSD Fisher’s test).

**Figure 5:**
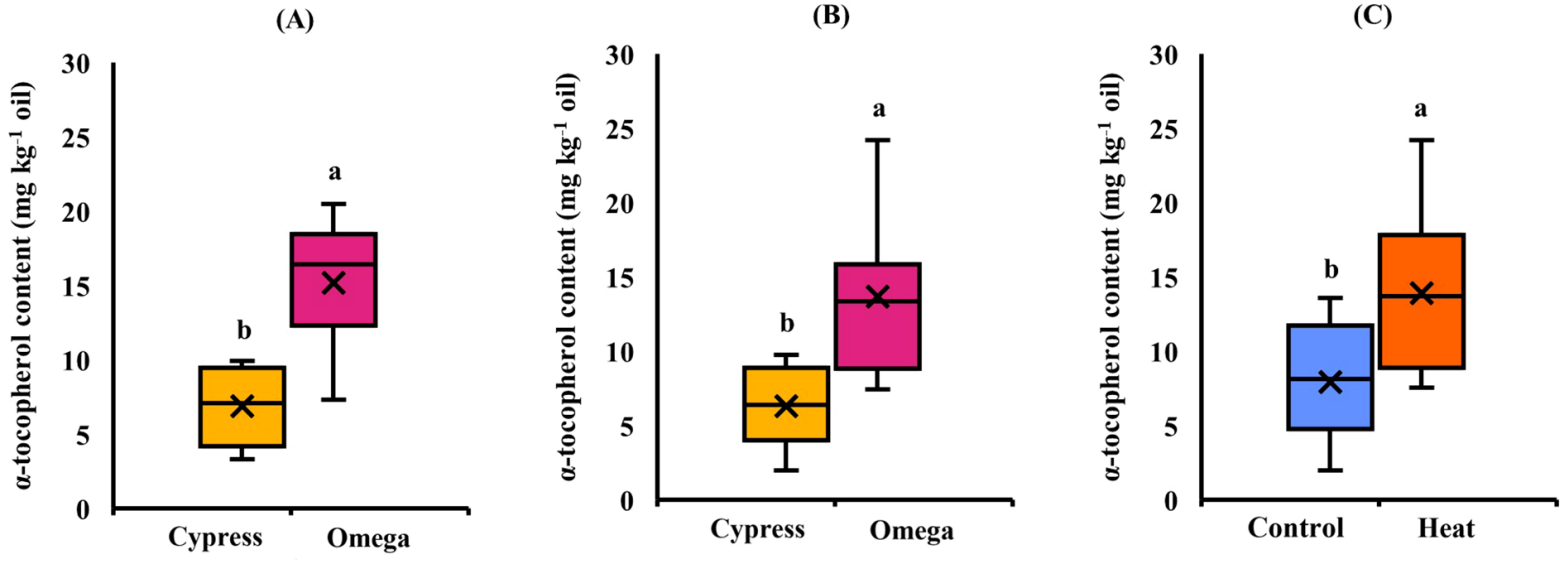
α-Tocopherol content in response to cultivar in EXP1 **(A)** and EXP2 **(B)** and in response to treatment in EXP2 **(C)**. Different letters: significantly different means (*p* ≤ 0.05, LSD Fisher’s test).

**Figure 6:**
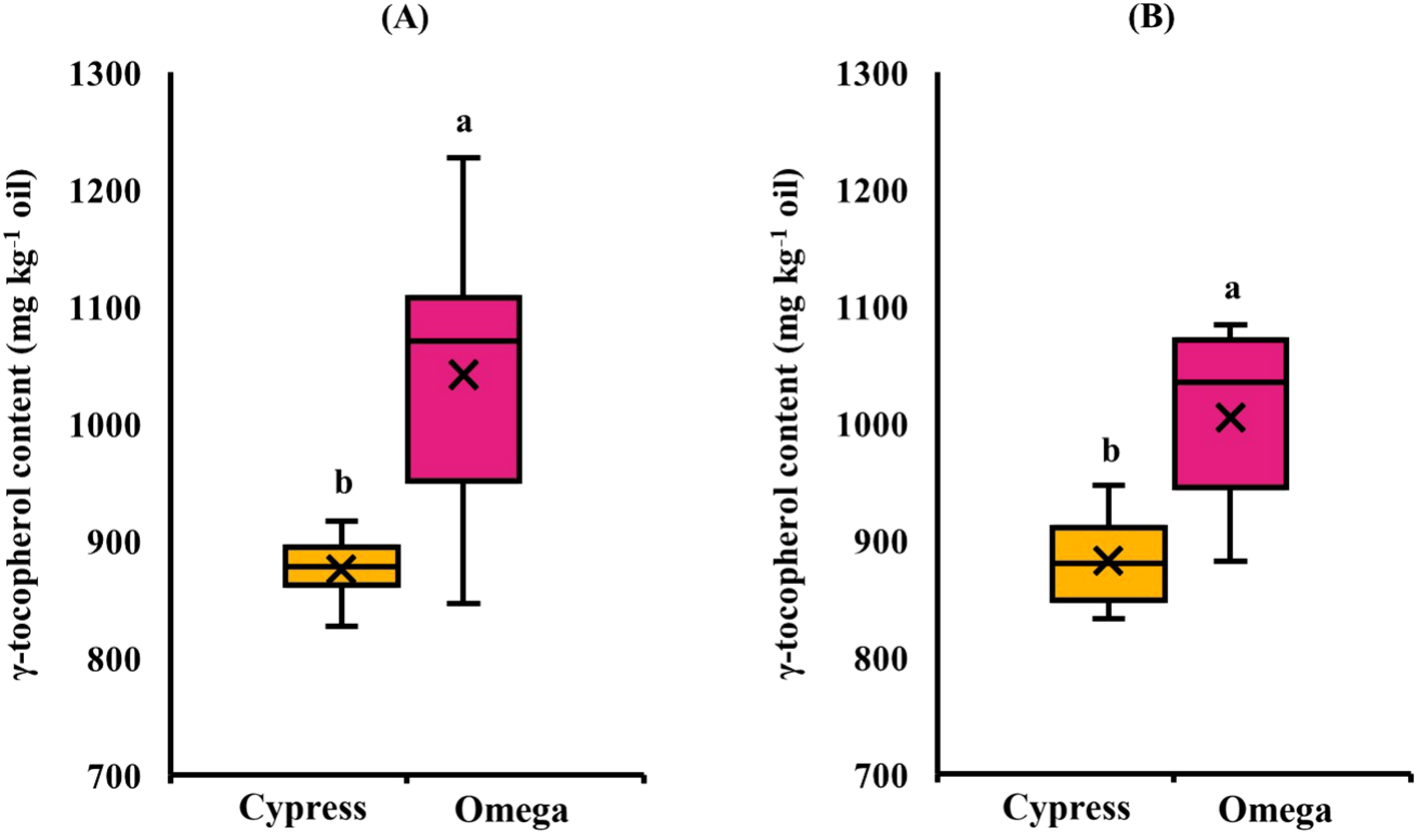
γ-Tocopherol content in response to cultivar in EXP1 **(A)** and EXP2 **(B)**. Different letters: significantly different means (*p* ≤ 0.05, LSD Fisher’s test).

In EXP1(**Table 5**), high temperature caused a significant (*p* ≤ 0.05) decrease in seed oil content, which was 31.4% in control and decreased to 28.3% in stressed plants, corresponding to a reduction of up to − 9.89% (**Figure 2A**). Regarding oil composition, the fatty acids C18:1 and C18:3 were significantly (*p* ≤ 0.05) affected by cultivar. C18:1 was also influenced by treatment, whereas C18:2 remained stable in response to all experimental factors. C18:1 was significantly higher in Omega (16.3%) than in Cypress (12.7%) (**Figure 3A**) and was higher in control plants (14.9%) than in stressed plants (14.1%) (**Figure 3B**). Cultivar Cypress showed higher C18:3 content (35.1%) compared with Omega (34.1%) (**Figure 4A**). Concerning tocopherols, Omega had the significantly (*p* ≤ 0.01) highest total tocopherol (1,065.8 mg kg^−1^ oil), α-tocopherol (15.2 mg kg^−1^ oil), and γ-tocopherol (1,041.8 mg kg^−1^ oil) contents compared with Cypress, in which total tocopherols were 893.9 mg kg^−1^ oil, α-tocopherol was 6.88 mg kg^−1^ oil (**Figure 5A**), and γ-tocopherol was 875.9 mg kg^−1^ oil (**Figure 6A**).

**Table 5:** ANOVA table with *F*-values and statistical significance for oil content, main fatty acids, tocopherol content, and tocopherol isoforms.

Source of variation	Oil content	C18:1	C18:2	C18:3	Total tocopherol	α-Tocopherol	γ-Tocopherol
EXP1							
Cultivar	0.20 ns	255.5^**^	1.89 ns	5.57^*^	20.1^**^	30.4^**^	18.9^**^
Treatment	6.59^*^	13.4^**^	1.74 ns	0.03 ns	0.10 ns	3.86 ns	0.15 ns
Cultivar × treatment	0.42 ns	1.78 ns	0.81 ns	0.25 ns	0.85 ns	0.02 ns	0.88 ns
EXP2							
Cultivar	1.14 ns	59.3^**^	8.92^*^	1.02 ns	12.9^**^	20.5^**^	12.5^**^
Treatment	8.89^*^	63.5^**^	12.9^**^	19.9^**^	0.61 ns	12.4^**^	0.31 ns
Cultivar × treatment	0.12 ns	6.65^*^	4.15 ns	0.02 ns	0.65 ns	0.41 ns	0.74 ns

^*^0.05 and ^**^0.01 indicate probability levels of significance (LSD Fisher’s test). *ns*, not significant.

In EXP2 (**Table 5**), seed oil content was also significantly (*p* ≤ 0.05) reduced by 11.6% under high temperature (31.2% in control and 27.6% in stressed plants) (**Figure 2B**). Regarding fatty acid content of seed oil, there was a significant interaction between cultivar and treatment (*p ≤* 0.05) for C18:1. The main factor cultivar significantly (*p* ≤ 0.05) affected C18:2, while high temperature influenced both C18:2 and C18:3. C18:1 was again higher in Omega (17.9%) than in Cypress (14.2%), and high temperature enhanced its content (14.4% in control and 18.6% in stressed plants); however, the increase due to heat treatment was more evident in Omega (+ 33.1%) than in Cypress (+ 20.2%) (**Figure 3C**). C18:2 was higher in Cypress (17.5%) than in Omega (16.1%) (**Figure 4B**) and increased under high temperature, with content in stressed plants of 17.3% compared with 16.0% in control plants (**Figure 4C**). In contrast, C18:3 content decreased in stressed plants (30.7%) compared with controls (34.3%) (**Figure 4D**). Regarding tocopherols, Omega had significantly (*p* ≤ 0.01) higher total tocopherol (1,028.2 mg kg^−1^ oil) content than Cypress (899.7 mg kg^−1^ oil), as well as higher content of the analyzed isoforms: α-tocopherol (13.7 mg kg^−1^ oil, **Figure 5B**) and γ-tocopherol (1,005.1 mg kg^−1^ oil, **Figure 6B**), whereas in Cypress they were 6.31 and 882.3 mg kg^−1^ oil, respectively. Finally, high temperature significantly (*p* ≤ 0.01) increased α-tocopherol content in stressed plants (13.9 mg kg^−1^ oil) compared with control plants (7.92 mg kg^−1^ oil), representing an increase of 75.8% (**Figure 5C**).

## 4 Discussion

Over recent years, the influence of global climate change has resulted in increased average temperatures, coupled with a rising frequency of extreme weather events, e.g., heat waves. Predictions for the coming years indicate that the warming trend will continue, with an increased likelihood of extreme heat episodes. In this scenario, the Mediterranean region has been identified as highly vulnerable, where the effects of climate change will be exacerbated. These effects will include heat waves and days when the maximum temperature exceeds 37°C (Lazoglou et al., 2024). Current predictions suggest that the agricultural sector will be particularly impacted by these phenomena, with repercussions including crop losses and reduced yield. Heat waves can be detrimental throughout the entire growth cycle of plants; however, reproductive stages are particularly heat-sensitive, with a lower threshold temperature compared to the vegetative phase, making seed set and maturation more susceptible. Temperatures above this threshold during the reproductive phase can interfere with pollen viability and grain formation, thus causing considerable yield losses (Hatfield et al., 2011; Kaushal et al., 2016; Prasad et al., 2017). With this knowledge, it becomes essential to identify varieties that can cope with high temperatures, producing economic yields even in areas expected to be most exposed to rising temperatures.

Literature reports yield losses under heat stress for camelina and other *Brassicaceae*, such as *Brassicaceae juncea*, *Brassicaceae napus* L., and *Brassicaceae rapa* (Angadi et al., 2000; Carmo-Silva and Salvucci, 2012; Elferjani and Soolanayakanahally, 2018). Other studies report reductions in yield components, such as the number of siliques (Elferjani and Soolanayakanahally, 2018) and seed weight (Ahmad et al., 2021; Aksouh-Harradj et al., 2006; Carmo-Silva and Salvucci, 2012). Nevertheless, in the present work, heat stress did not cause detectable yield losses. The only yield component that was significantly impacted by heat was the number of siliques, which increased when stress was applied at EXP1, in line with previous observations (Angadi et al., 2000); however, there was no effect on final yield (**Table 4**). It is possible that stressed plants produced more siliques as a compensation strategy in response to stress; however, the observed decline in net photosynthesis likely reduced the availability of photosynthates, making it probable that these extra siliques were effectively seedless.

Results of the morphological and physiological measurements carried out during the camelina growth cycle suggested a limited impact of the imposed stress conditions, compared to a more pronounced effect of cultivar (**Tables 2**, **3**). Despite the imposed temperatures of 35 °C and 40 °C (EXP1 and EXP2), which correspond to the threshold levels usually observed for crops during the reproductive stage (Prasad et al., 2017), the only parameters affected by heat were plant height and net photosynthesis in EXP1, and the number of leaves in EXP2. The limited effects observed on gas exchanges can be ascribed to the GATA genes, a heat-sensitive family present in camelina that is involved in heat stress resistance (Kim, 2024; Zhu et al., 2023). The calculated net variation in EXP1 revealed that high temperature caused a reduction in net photosynthesis, together with an increase in stomatal conductance. These changes can be attributed to the regulation of stomata opening to decrease the internal temperature and to the slowing down of photosynthetic activity due to stress. Considering that photosynthetic activity of siliques is directly responsible for yield determination and, by generating seed assimilates, is a major source of nutrients for developing seeds (Bennett et al., 2011; Hua et al., 2012), it is evident that plants in EXP1 were able to recover from heat and the reduced photosynthetic activity of siliques, showing no difference in final seed yield compared to controls. In EXP2, no significant effects on gas exchanges were observed, presumably because the plants were already undergoing senescence, as indicated by the delta values of net photosynthesis, which decreased in both control and stressed plants, although the decrease was greater in stressed individuals.

The absence of yield loss observed in both experiments in this work may have several explanations. Firstly, stress may have been imposed after yield components were physiologically determined, so high temperature did not have a detrimental effect on seed production, as also observed by Aksouh-Harradj et al. (2006). Another possible reason is that heat was applied at a level and/or for a duration that allowed camelina to tolerate it; indeed, Smith and Lu (2024) proposed that the duration of heat affects its impact on seed yield. It is also possible that the night temperature set at 18 °C helped the plants to recover from heat, as suggested by Chen et al. (2021). Finally, it should be considered that the present work was carried out under controlled conditions, and only one abiotic stress (heat) was imposed. In open-field conditions, abiotic stresses such as heat and drought often occur simultaneously, and biotic stresses may also be present.

Seed weight, particularly the 1,000-seed weights, is a key characteristic of camelina due to its generally low values, ranging from 0.7 to 1.8 g (Zanetti et al., 2021). In this experiment, heat did not affect this trait, as also observed by Chen et al. (2021). In contrast, cultivar had a significant effect, with Cypress showing higher seed weight, suggesting that this attribute is mainly genetically determined.

Unlike seed yield, a reduction in seed oil content was observed in both experiments, significant but limited in magnitude (− 9.89% in EXP1, **Figure 2A**; and − 11.6% in EXP2, **Figure 2B**). This finding is in line with previous work on other *Brassicaceae* (Canvin, 1965; Elferjani and Soolanayakanahally, 2018; Hossain et al., 2019) and can be ascribed to the trends in net photosynthesis described above, as seed lipid synthesis relies on photosynthates provided by the silique wall (Hua et al., 2012).

Fatty acids are among the main components of camelina oil, and their contents were affected by heat stress, as expected from the literature (Baud and Lepiniec, 2010; Righini et al., 2019). Elevated temperatures typically induce changes in fatty acid composition, and earlier studies have generally observed a decrease in trienoic fatty acids (C16:3 and C18:3) and an increase in dienoic (C18:2) species. The relative abundance of different desaturated fatty acid species and their derivatives in plants is partly regulated by the activity of fatty acid desaturases (FADs). FADs introduce double bonds into fatty acids (C18:2 and C18:3) at specific positions, shaping the properties of the molecule and influencing cellular membrane characteristics. In plants, FADs desaturate polar glycerolipids locally. In extraplastidic glycerolipid classes, FAD2 converts oleic acid (C18:1) to linoleic acid (C18:2), which is further desaturated to linolenic acid (C18:3) by FAD3. For plastidic glycerolipid classes, FAD6 desaturates C18:1 to produce C18:2, while FAD7 and FAD8 redundantly catalyze the conversion of C18:2 to C18:3 (Li-Beisson et al., 2013). Therefore, regulation of fatty acid saturation by FADs is an important mechanism for plants to adapt to elevated temperatures. Evidence suggests that, in response to temperature, FAD activity is regulated primarily at the posttranscriptional level, involving modulation of FAD protein half-life via a combination of *cis*-acting degradation signals and the ubiquitin-proteasome pathway (O’Quin et al., 2010). Reflecting the network of activities that determine seed oil fatty acid composition, the effects of the two heat treatment experiments differed. In EXP1, heat stress decreased C18:1 content (**Figure 3B**), as also observed by Elferjani and Soolanayakanahally (2018). Conversely, in EXP2, where stress was applied later, C18:3 was reduced by heat (**Figure 4D**), while C18:1 and C18:2 were increased (**Figures 3C**, **4C**), consistent with previous works on camelina (Nadakuduti et al., 2023; Righini et al., 2019). Canvin (1965) reported that heat deactivates the enzymes responsible for converting C18:1 to C18:2 and C18:3. The temperature response of monounsaturated fatty acids (C18:1) is often species- and tissue-specific, reflecting the regulatory control of stearoyl-acyl carrier protein desaturase (SAD), which catalyzes the first desaturation step leading to C18:1. Confounding responses of C18:1 to elevated temperatures likely reflect the combined regulatory control of acyl-ACP thioesterase genes FATB and FATA. Acyl-ACP-thioesterase Type B (FATB) competes with SAD for the 18:0-Acyl Carrier Protein (ACP) substrate, and Acyl-ACP-thioesterase Type A (FATA) competes with FAD2 for the 18:1-ACP substrate (Byfield and Upchurch, 2007). The effects observed in EXP2 suggest that further desaturation of fatty acids is disfavored under heat stress, and lower C18:3 content has been associated with heat tolerance (Nadakuduti et al., 2023). The different behaviors of fatty acids observed in the two experiments can be explained by the fact that C18:2 and C18:3 accumulate at a later stage in camelina compared to C18:1 (Rodríguez-Rodríguez et al., 2013), so heat in EXP2 had a greater impact than in EXP1.

Considering the antioxidant activity of tocopherols, they should be regarded as a distinctive trait of camelina oil, on par with fatty acids. Indeed, plants capable of coping with stress have higher tocopherol content (Chen et al., 2022). Tocopherols localize in membranes, where they associate with polyunsaturated fatty acids (PUFAs) and influence properties such as membrane permeability and stability. They play an important role in plant stress responses, e.g., *Arabidopsis* mutants lacking a functional homogentisate phytyl transferase—the first committed enzyme of the tocopherol biosynthetic pathway—display a phenotype only under temperature stress. Reduced tocopherol content affects the production of C18:3 in the ER, rather than in chloroplastic lipid metabolism, demonstrating how chloroplast-derived metabolites influence extraplastidic lipid metabolism as part of the overall cellular temperature response (Maeda et al., 2008).

In the present study, γ-tocopherol was found to be under genetic control, and total tocopherol content showed the same pattern. This is because in camelina oil, γ-tocopherol accounts for more than 90% of total tocopherols (Zubr and Matthaus, 2002). Conversely, α-tocopherol was significantly increased by heat stress in EXP2 (+ 75.8%, **Figure 5C**), as reported in other studies (Britz and Kremer, 2002; Chennupati et al., 2011; Sadiq et al., 2019). During heat stress, ROS are produced at higher levels, threatening cell survival. Consequently, tocopherols accumulate in greater amounts to protect photosystem II, reduce ROS levels, and control lipid peroxidation (Falk and Munné-Bosch, 2010; Lushchak and Semchuk, 2012; Munné-Bosch and Alegre, 2002). As previously stated, γ-tocopherol did not show the same behavior. This may be due to two reasons. The first is that α-tocopherol has higher antioxidant activity in plant tissues than the γ-isoform (Munné-Bosch and Alegre, 2002). The second is related to the activity of the enzyme γ-tocopherol methyltransferase, which converts γ-tocopherol to α-tocopherol (Hunter and Cahoon, 2007). It has been suggested that γ-tocopherol methyltransferase activity is enhanced by elevated temperature (Chennupati et al., 2011; Koch et al., 2002). Moreover, Gálvez-Valdivieso et al. (2011) reported that TMT1, the gene encoding γ-tocopherol methyltransferase, has a temperature optimum of around 40 °C, which may explain why the increase in α-tocopherol was observed only in EXP2.

Lastly, in the present study, a significant effect of cultivar was observed for many traits. Omega had the highest content of C18:1 (**Figure 3A**), as previously reported by Alberghini et al. (2022), and an increased tocopherol content. Cypress was confirmed as the best cultivar when considering seed yield, 1,000-seed weight, and C18:3 content, in agreement with previous studies by Zanetti et al. (2017) and Alberghini et al. (2022).

## 5 Conclusion

The present work addressed the response of the camelina cultivars Cypress and Omega to heat stress during two periods of the reproductive phase, namely the end of flowering and the end of silique formation. The cultivar was the main driver of the morphological and physiological parameters, with a slight influence from either the earlier or late heat treatment, with the only exception being the reduction in net photosynthesis under earlier stress. Although both cultivars were able to maintain their seed yield under the tested conditions, a modest decline in seed oil content was observed; thus, although seed quality was slightly affected, camelina generally tolerated the imposed stress. Fatty acids were mostly altered by high temperature at the end of silique formation, with an increase in C18:1 and C18:2 coupled with a reduction in C18:3. α-Tocopherol was greatly enhanced by the later heat stress, suggesting that it plays a major role in high-temperature resistance at a more advanced developmental stage. On the other hand, it is suggested that, under earlier heat stress, other molecules (i.e., glutathione, ascorbate, carotenoids) are necessary for the antioxidant defense of tissues. Lastly, considering the performance of the two cultivars, Cypress had the best agronomic performance in all conditions, while Omega showed an improved tocopherol content. The authors suggest that future work could complement the results of the present study by simulating intermittent heat stress and including a larger panel of camelina varieties, which could help to better understand how this species responds to stress.

## Data Availability

The raw data supporting the conclusions of this article will be made available by the authors, without undue reservation.
